# 

**Published:** 1911-12

**Authors:** 


### The Library of the Bristol Medico-Chirurgical Society.

---

*The following donations have been received since the publication of the List in September.*

*November 30th,* 1911.

| | |
|---|---|
| J. Paul Bush, C.M.G. .. .. .. .. | 4 volumes. |
| J. A. Nixon, M.B. (1) .. .. .. .. | 1 ,, |
| R. Shingleton Smith, M.D. (2) .. .. .. | 1 ,, |
| L. M. Griffiths (3) .. .. .. .. .. | 1 ,, |
| F. Wallis Stoddart .. .. .. .. .. | 12 ,, |

Framed pictures have been received from Dr. J. Michell Clarke and Mr. L. M. Griffiths, and pamphlets have been presented by Mr. Paul Bush and Mr. L. M. Griffiths.

### EIGHTY-SECOND LIST OF BOOKS.

The titles of books mentioned in previous lists are not repeated.

The figures in brackets refer to the figures after the names of the donors, and show by whom the volumes were presented. The books to which no such figures are attached have either been bought from the Library Fund or received through the *Journal*.

**Alderson, W. E.** .. *Dental Anæsthetics* .. .. .. .. .. .. .. 1911

**Allbutt and H. D. Rolleston, Sir C.** [Eds.] *A System of Medicine.* Vol. IX 1911

**Backer, F. de** .. *Lourdes et les Médecins* .. .. .. .. .. (3) 1905

**Ballantyne, J. W.** [Ed.] *Quinquennium of Medicine and Surgery* (1906–10) 1911

**Bidwell, L. A.** .. *Minor Surgery* .. .. .. .. .. .. .. .. .. .. 1911

**Cheyne, Sir W. W.** *Tuberculous Diseases of Bones and Joints* 2nd Ed. 1911

**Colyer, J. F.** .. .. *Dental Surgery and Pathology* .. .. .. .. .. 1910

**Ely, L. W.** .. .. *Joint Tuberculosis* .. .. .. .. .. .. .. .. 1911

**Fenwick, W. S.** .. *Dyspepsia* .. .. .. .. .. .. .. .. .. 1910
**Firth, R. H.** .. .. *Military Hygiene* .. .. .. .. .. .. .. .. 1908
**Fordyce, A. D.** .. *The Care of Infants and Young Children* .. 1911
**Groves, E. W. H.** .. *A Synopsis of Surgery* .. .. .. .. 3rd Ed. 1911
**H., A. A.** .. .. *Where the World Ends* .. .. .. .. .. (2) 1911
**Hertz, A. F.** .. .. *The Sensibility of the Alimentary Canal* .. 1911
**Hewer, Mrs. J. L.** .. *Our Baby* .. .. .. .. .. .. 13th Ed. 1911
**Jefferys and J. L. Maxwell, W. H.** *Diseases of China* .. .. .. .. 1910
**Ker, C. B.** .. .. *A Manual of Fevers* .. .. .. .. .. .. .. .. 1911
**Lockwood, C. B.** .. *Clinical Surgery* .. .. .. .. .. 2nd Ed. [1911]
**Lyle, H. W.** .. .. *Manual of Physiology* .. .. .. .. .. .. 1911
**Maxwell, W. H. Jefferys and J. L.** *Diseases of China* .. .. .. .. 1910
**Mennell, J. B.** .. *Treatment of Fractures by Mobilisation and Massage* .. .. .. .. .. .. .. .. .. .. 1911
**Mercier, C. A.** .. *Conduct and its Disorders* .. .. .. .. .. 1911
**Miles, A. Thomson and A.** *Manual of Surgery.* Vol. I .. 4th Ed. 1911
**Milligan and W. Wingrave, W.** *Diseases of the Ear* .. .. .. .. 1911
**Mortimer, J. E.** .. *Anæsthesia and Algesia* .. .. .. .. .. .. 1911
**Rolleston, Sir C. Allbutt and H. D.** *A System of Medicine.* Vol. IX 1911
**Short, A. R.** .. .. *The New Physiology in Surgical and General Practice* .. .. .. .. .. .. .. .. .. .. 1911
**Swanzy and L. Werner, Sir H. R.** *Diseases of the Eye* 10th Ed. 1912
**Thomson and A. Miles, A.** *Manual of Surgery.* Vol. I 4th Ed. 1911
**Thomson, St. C.** .. *Diseases of the Nose and Throat* .. .. .. 1911
**Tuckett, I. L.** .. .. *The Evidence for the Supernatural* .. .. .. 1911
**Werner, Sir H. R. Swanzy and L.** *Diseases of the Eye* .. 10th Ed. 1912
**Wingrave, W. Milligan and W.** *Diseases of the Ear* .. .. .. .. .. 1911
**Wolff-Eisner, A.** *Clinical Immunity and Sero-Diagnosis* (Trans. by R. W. Matson) .. .. .. .. .. .. .. 1911

### TRANSACTIONS, REPORTS, JOURNALS, &c.

American Journal of the Medical Sciences, The .. .. Vol. CXLI 1911
American Journal of Physiologic Therapeutics .. .. Vol. I 1910–11
Birmingham Medical Review, The .. .. N.S., Vols. XV, XVI 1910
British Medical Journal, The .. .. .. .. .. .. .. .. Vol. I for 1911
Edinburgh Medical Journal .. .. .. .. .. .. .. N.S., Vol. VI 1911
Edinburgh Obstetrical Society's Transactions .. .. Vol. XXXVI 1911
Gazette des Hôpitaux .. .. .. .. .. .. .. .. .. .. .. .. .. 1910
Glasgow Medical Journal, The .. .. .. .. .. .. Vol. LXXV 1911
Hospital, The .. .. .. .. .. .. .. .. .. .. .. Vol. XLIX 1911
Journal of Experimental Medicine, The .. .. .. Vol. XIII 1911
Lancet, The .. .. .. .. .. .. .. .. .. .. .. .. Vol. I for 1911
Northumberland and Durham Medical Journal, The .. .. .. .. 1909–10
Ophthalmological Society's Transactions .. .. .. Vol. XXX 1910
Practitioner, The .. .. .. .. .. .. .. .. .. .. Vol. LXXXVI 1911
Progressive Medicine .. .. .. .. .. .. .. .. .. Vol. II 1911
Universities in Great Britain, Reports from, for 1909–10 .. (1) 1911

*A Handbook of Climatic Treatment, including Balneology.* London, 1906. Macmillan. 552 pp. 8vo.

*Davos as Health Resort.* Introduction by W. R. Huggard. Davos, 1907. 320 pp., 27 pl. 8vo.

"The Influence of High Altitudes," *Practitioner,* 1908, lxxxi. 95.

"Classification of Cases in Pulmonary Tuberculosis," *Brit. M. J.*, 1909, ii. 1403.

### Local Medical Notes.

**University of Bristol.**—The conferring of degrees took place on October 20th, when, amongst others, Honorary Degrees were conferred on Professor Alfred Marshall, Sir William Ramsay, K.C.B., Professor A. P. Chattock, and Professor Wertheimer. In the evening the dinner of the Association of Alumni took place.

The Medical School Dinner took place on November 21st, under the presidency of Mr. C. E. S. Flemming. The guest of the evening was Mr. Howard Marsh, Regius Professor of Surgery, University of Cambridge.

### EXAMINATION RESULTS.

F.R.C.S. Eng., Primary Examination.—A. E. Iles, W. Salisbury.

Conjoint Board.—*Chemistry and Physics:* Louisa M. Lister. *Midwifery:* G. S. Applegate. *Surgery:* R. F. Bolt.*

L.D.S., R.C.S. Eng.—*Dental Mechanics:* W. J. Milton, W. Adams. *Dental Metallurgy:* W. Adams. *Final Examination:* (*Part I*) P. W. Flook; (*Part II*) E. J. Handford, W. A. Slade, C. H. Wilson.

### APPOINTMENTS.

**Bristol Royal Infirmary.**—S. H. Kingston, M.B., Ch.B. Bristol, has been appointed Senior Resident Officer.

**Bristol General Hospital.**—The following have been re-appointed:—G. Parker, M.A., M.D. Cantab., Honorary Physician; Newman Neild, M.B., Ch.B. Vict., Assistant Physician; F. G. Bergin, M.R.C.S., L.R.C.P., Skiagraphist.

* Completes examination.

Dr. Percival Mackie, of the India Medical Service, has been appointed Chemical Examiner Analyst, and Bacteriologist to the Government of the United and Central Provinces; headquarters, Agra.

**Medical Library Report, 1911.**—It will be of interest briefly to mention a few facts in connection with the past history of the Medical Library. The Library was first opened on January 5th, 1891, in a room placed at the disposal of the Medico-Chirurgical Society by the Literary and Philosophic Club at 28 Berkeley Square. Here the Library remained until July 5th, 1893, when the collection of books owned by the Society were transferred to a handsome, spacious room, especially constructed for a library, in the new Medical School buildings of the University College. Here the collection of books of the conjoined libraries of the Society and the University of Bristol has remained until the present year, when for a second time the books forming the Bristol Medical Library have been removed into another building, the Council of the University having fitted up the east wing of the old Blind Asylum for the accommodation of the Library.

The books, instead of being located in one large room, will now be distributed between four medium-sized rooms, one being assigned to bound periodicals. These changes will necessitate an entire re-arrangement of the books and the making of a fresh catalogue.

The Council of the University have promised to make a substantial yearly money grant to the Library Committee for the purchase of books.

A handsome pitchpine book-case of large dimensions has been presented to the Society by Dr. P. Watson-Williams; framed pictures and other donations of this kind would be gratefully accepted by the Library Committee on behalf of the Society.

### INDEX.

Amyotonia congenita, 380.
Arsenic compounds and optic atrophy, 79.
Arterial pressures, The measurement of, 165.
Arthritis, multiple, caused by tuberculin, 348.
Ataxia in a child, Case of—Dr. E. C. Williams, 163.
Auricular fibrillation—Dr. C. E. K. Herapath, 148.

Beddoe, J., Obituary notice of, 280, 284.
Bentley, A. J. M., Obituary notice of, 197.
Blacker, E., Obituary notice of, 98.
Bladder, Treatment of stone in the, 169.
Blood pressures, Clinical interpretation of, 168.
Blood pressures, Normal and abnormal, 168.

**Books, Reviews of—**

After-results of operations on the pelvic organs—E. Giles, 361.
Allbutt and Dr. H. D. Rolleston, Sir C. [Eds.]—A system of medicine, 81, 259.
Alpinism, Alcohol and—Dr. L. Schnyder, 176.
Anatomy, Surgical—C. R. Whitaker, 266.
Arderne, J.—Treatises of fistula in ano, etc., 263.
Association of American Physicians, Transactions of the, 364.
Ballantyne, Dr. J. W. [Ed.]—Quinquennium of medicine and surgery, 356.
Bandaging, Pye's elementary, 269.
Berkeley and V. Bonney, Drs. C.—Gynæcological surgery, 265.
Bidwell, L. A.—Intestinal surgery, 269.
Billington, W.—Movable kidney, 263.
Bone, Disease in, and its detection by X-rays—E. H. Shenton, 359.
Bonney, Drs. C. Berkeley and V.—Gynæcological surgery, 265.
Bosanquet and J. W. H. Eyre, Drs. W. C.—Serums, vaccines and toxines, 83.
Bresler, Dr. J.—Treatment of syphilis by the Ehrlich-Hata remedy, 276.
Brown, Dr. W. L.—Physiological principles of treatment, 270.
Castellani and A. J. Chalmers, Drs. A.—Tropical medicine, 80.
Catalogue (Index-) of the library of the Surgeon-General's office, 277.

**Books, Reviews of** (*continued*)—

Chalmers, Drs. A. Castellani and A. J.—Tropical medicine, 80.
Charpentier, Dr. A. E. L.—Hæmoglobinuria, 268.
Chemistry of synthetic drugs—P. May 354.
Clarke, W. B.—Handbook of the surgery of the kidneys, 362.
Clinical pathology in practice—Dr. T. J. Horder, 179.
College of Physicians of Philadelphia, Transactions of the, 266, 365.
Dispensing made easy—Dr. W. G. Sutherland, 275.
Donahoe, M. F.—A manual of nursing, 364.
Drugs, Chemistry of synthetic—P. May, 354.
Ear, Operations on the—B. Heine, 273.
Edmunds, Dr. A.—Glandular enlargement, 264.
Education, Some considerations of medical —Dr. S. S. Sprigge, 178.
Emersen, Drs. R. W. Levett and H. C.—Infantile paralysis in Massachusetts, 268.
Evolution and heredity, Phases of—Dr. D. B. Hart, 259.
Eyre, Drs. W. C. Bosanquet and J. W. H.—Serums, vaccines and toxines, 83.
Fein, Dr. J.—Hints in rhinology and laryngology, 86.
Fevers, Manual of—Dr. C. B. Ker, 358.
Fishberg, M.—The Jews, 257.
Fistula in ano, etc., Treatises on—J. Arderne, 263.
Fortescue-Brickdale, Dr. J. M.—A practical guide to the newer remedies, 172.
Fractures and separated epiphyses—A. J. Walton, 261.
Giles, Dr. A. E.—A study of the after-results of abdominal operations on the pelvic organs, 361.
Glaister, Dr. J.—Public health, 274.
Glandular enlargement—Dr. A. Edmunds, 264.
Gynæcological surgery—Drs. C. Berkeley and V. Bonney, 265.
Hæmoglobinuria—Dr. A. E. L. Charpentier, 268.
Haig, Dr. A.—Uric acid in the clinic, 272.
Hart, Dr. D. B.—Phases of evolution and heredity, 259
Hartley, Sir R. D. Powell and Dr. P. H.-S. —Diseases of the lungs and pleuræ, 358.
Hay fever—Dr. E. S. Yonge, 274.
Heine, B.—Operations on the ear, 273.
Heredity, Phases of evolution and—Dr. D. B. Hart, 259.
Herman, Dr. G. E.—Difficult labour, 85.
Hernia—R. W. Murray, 269.
Histology, Essentials of—Dr. E. A. Schafer, 361.

**Books, Reviews of** (*continued*)—
History of medicine—Dr. M. Neuburger, 256.
Hollander, Dr. B.—The mental symptoms of brain disease, 260.
Horder, Dr. T. J.—Clinical pathology in practice, 179.
Hunt and A. Seidell, R.—Studies on thyroid, 271.
Hygiene and public health—Drs. L. C. Parkes and H. R. Kenwood, 363.
Hypnotism, Introduction to the study of—Dr. H. E. Wingfield, 86.
Hypnotism, Suggestive power of—Dr. F. Winslow, 87.
Infantile paralysis in Massachusetts—Drs. R. W. Levett and H. C. Emersen, 268.
Infants, Acute intestinal toxæmia in—Dr. R. Vincent, 361.
Infectious diseases in schools, Code of rules for the prevention of, 275.
Intestinal surgery—L. A. Bidwell, 269.
Intestinal toxæmia in infants, Acute—Dr. R. Vincent, 361.
Jews, The—M. Fishberg, 257.
Johns Hopkins hospital reports, 177.
Joints and spine, Diseases of—H. Marsh, 84.
Kelynack, Dr. T. N.—Medical examination of schools and scholars, 175.
Kenwood, Drs. L. C. Parkes and H. R.—Hygiene and public health, 363.
Ker, Dr. C. B.—Manual of Fevers, 358.
Kidd, F.—Urinary surgery, 174.
Kidney, Movable—W. Billington, 263.
Kidneys, Surgery of the—W. B. Clarke, 362.
Labour, Difficult—Dr. G. E. Herman, 85.
Lamb, Dr. W.—Guide to the diseases of the throat, nose and ear, 273.
Laryngology, Hints in—Dr. J. Fein, 86.
Levett and H. C. Emersen, Drs. R. W.—Infantile paralysis in Massachusetts, 268.
Loose-leaf medical pocket-book, 84.
Lungs and pleuræ, Diseases of the—Sir R. D. Powell and Dr. P. H.-S. Hartley, 358.
Macilwaine, S. W.—Medical revolution, 365.
Maddox, Dr. E. E.—Golden rules of refraction, 363.
Marie, Dr. A. [Ed.]—Traité international de psychologie pathologique, 353.
Marsh, H.—Diseases of the joints and spine, 84.
May, P.—The chemistry of synthetic drugs, 354.
Medical annual, The, 267.
Medicine, History of—Dr. M. Neuburger, 256.
Medicine, System of—Sir C. Allbutt and Dr. H. D. Rolleston [Eds.], 81, 259.
Meningitis, sinus thrombosis and abscess of the brain—Dr. J. Wyllie, 179.
Mental symptoms of brain disease, The—Dr. B. Hollander, 260.
Merck's annual report, 277.
Morison, R.—An introduction to surgery, 269.
Moullin, Dr. C. M.—Enlargement of the prostate, 270.
Murray, R. W.—Hernia, 269.
Neuburger, Dr. M.—History of medicine, 256.
Neumann, Dr. H.—Otitic cerebellar abscess, 273.
Nose and throat, Diseases of the—Dr. St. C. Thomson, 360.
Nursing, Manual of—M. F. Donahoe, 364.

**Books, Reviews of** (*continued*)—
Obstetrics, Practical—Drs. E. H. Tweedy and G. T. Wrench, 86.
Otitic cerebellar abscess—Dr. H. Neumann, 273.
Parkes and H. R. Kenwood, Drs. L. C.—Hygiene and public health, 363.
Pelvic organs, After-results of operations on the—Dr. A. E. Giles, 361.
Pharmacy, Chronicles of—A. C. Wootton, 171.
Philadelphia, Transactions of the College of Physicians of, 266, 365.
Physiological principles of treatment—Dr. W. L. Brown, 270.
Physiology in surgical and general practice, The new—Dr. A. R. Short, 357.
Pleuræ, Diseases of the lungs and—Sir R. D. Powell and Dr. P. H.-S. Hartley, 358.
Powell and Dr. P. H.-S. Hartley, Sir R. D. —Diseases of the lungs and pleuræ, 358.
Prescriber's companion, A —Dr. T. D. Savill, 176.
Prostate, Enlargement of—Dr. C. M. Moullin, 270.
Psychologie pathologique, Traité de—Dr. A. Marie, 353.
Public health (Catechism series), 275.
Public health, Text-book of—Dr. J. Glaister, 274.
Pye's elementary bandaging, 269.
Quinquennium of medicine and surgery, 356.
Refraction, Golden rules of—Dr. E. E. Maddox, 363.
Remedies, Guide to the newer—Dr. J. M. Fortescue-Brickdale, 172.
Remedies, New and non-official, 363.
Revolution, Medical—S. W. Macilwaine, 365.
Rhinology, Hints in—Dr. J. Fein, 86.
Rolleston, Sir C. Allbutt and Dr. H. D. [Eds.]—A system of medicine, 81, 259.
Salvarsan, Treatment of syphilis by—Dr. J. Bresler, 276.
Sanatorium, Le village, 270.
Savill, Dr. T. D.—A prescriber's companion, 176.
Schafer, Dr. E. A.—Essentials of histology, 361.
Schnyder, Dr. L.—Alcohol and alpinism 176.
Schofield, Dr. A. T.—How to keep fit, 364.
Schools and scholars, Medical examination of—Dr. T. N. Kelynack, 175.
Seidell, R. Hunt and A.—Studies on thyroid, 271.
Serums, vaccines and toxines—Drs. W. C. Bosanquet and J. W. H. Eyre, 83.
Shenton, E. H.—Disease in bone and its detection by the X-rays, 359.
Short, Dr. A. R.—The new physiology in surgical and general practice, 357.
Sickness and invalidity bill, 180.
Sleeping sickness commission, Reports of the, 84.
Smithsonian report, 87, 276.
Sprains and allied injuries of joints—Dr. R. H. A. Whitelocke, 362.
Sprigge, Dr. S. S.—Some considerations of medical education, 178.
Surgery, Introduction to—R. Morison, 269.
Sutherland, Dr. W. G.—Dispensing made easy, 275.
Thomson, Dr. St. C.—Diseases of the nose and throat, 360.
Thomson, J. A.—Outlines of zoology, 265.

**Books, Reviews of** (*continued*)—
Throat, Diseases of the nose and—Dr. St. C. Thomson, 360.
Throat, nose and ear, Diseases of the—Dr. W. Lamb, 273.
Thyroid, Studies on—R. Hunt and A. Seidell, 271.
Treatment, Physiological principles of—Dr. W. L. Brown, 270.
Tropical medicine—Drs. A. Castellani and A. J. Chalmers, 80.
Tweedy and G. T. Wrench, Drs. E. H.—Practical obstetrics, 86.
Urinary surgery—F. Kidd, 174.
Uric acid in the clinic—Dr. A. Haig, 272.
Vincent, Dr. R.—Acute intestinal toxæmia in infants, 361.
Walton, A. J.—Fractures and separated epiphyses, 261.
Watering places and sanatoria, Medical guide to, 268.
Whitaker, C. R.—Surgical anatomy, 266.
Whitelocke, Dr. R. H. A.—Sprains and allied injuries of joints, 362.
Wingfield, Dr. H. E.—An introduction to the study of hypnotism, 86.
Winslow, Dr. F.—The suggestive power of hypnotism, 87.
Wootton, A. C.—Chronicles of pharmacy, 171.
Wrench, Drs. E. H. Tweedy and G. T.—Practical obstetrics, 86.
Wyllie, Dr. J.—Meningitis, sinus thrombosis and abscess of the brain, 179.
X-rays detection of bone disease—E. H. Shenton, 359.
Yonge, Dr. E. S.—Hay fever and paroxysmal sneezing, 274.
Zoology, Outlines of—J. A. Thomson, 265.
Brachial neuritis, Herpes and—G. M. Smith, 194, 332.
Bristol Medical Library—L. M. Griffiths, 335.
Bristol Medical Library, 371, 384.
Bristol, Medical worthies of, in the seventeenth century—Dr. G. Parker, 201.
Bristol Medico-Chirurgical Society, 95, 191, 378.
British Medical Association, Birmingham meeting, 278.

Cardiac arrhythmia — Dr. C. Coombs, 321.
Cataract, Operative treatment of, 77.
Cerebro-spinal meningitis in Bristol, Outbreak of, 96.
Cerebro-spinal syphilis—Dr. J. M. Clarke, 1.
Cervical ribs, 348.
Charles, Dr. J. R.—Report on medicine, 345.
Chorea, The nature and treatment of—Dr. C. Coombs, 51.
Clarke, Dr. J. M.—The Long Fox lecture on cerebro-spinal syphilis, 1.
Conjunctivitis, Action of astringents in, 80.
Coombs, Dr. C.—Cardiac arrhythmia, 321; The nature and treatment of chorea, 51.
Cotton, Dr. W.—Design for an elementary radiographic camera, 118.
Cross, F. R.—Defects in the visual field, 33.
Davies, Dr. D. S., 281.
Dew, H. R., Obituary notice of, 287.
Duodenum, The end-results of operations on the stomach and—Dr. A. R. Short, 194, 220.

Editorial notes, 88, 180, 278, 366.
Education, Medical, and the Board of Education, 367.

Family type of general œdema, 192.
Filaria Loa, Case of, 381.
Fortescue-Brickdale, Dr. J. M.—Report on medicine, 165.
Fox (Long) Lecture, The, on cerebro-spinal syphilis—Dr. J. M. Clarke, 1.
Furunculosis—Dr. L. S. Smith, 157.

Gastric cancer, Ferment test for, 96.
Grace, E. M., Obituary notice of, 197.
Griffiths, L. M.—The Bristol Medical Library, 335.
Groves, E. W. H.—On the resection of the posterior spinal nerve roots, 105, 191.

Herapath, Dr. C. E. K.—Auricular fibrillation, 148.
Herpes and brachial neuritis—G. M. Smith, 194, 332.
Household syphilis — Dr. J. A. Nixon, 301.
Huggard, W. R., Obituary notice of, 382.
Hypernephroma of the liver, 380.

Immunity in the tuberculosis, 346.
Infantile paralysis in Bristol, Outbreak of, 96.
Infants, acquired syphilis in, Household syphilis, with observations on—Dr. J. A. Nixon, 301.
Insurance Bill, Sickness and, 180.

Johnson, P. P., Obituary notice of, 99.
*Johnson*, Boswell's *Life of*, Medical aspect of—Dr. B. M. H. Rogers, 125.

Kyle, H. G.—Report on surgery, 169.

Lansdown, R. G. P.—Report on surgery, 73.
Laryngological section of the Royal Society of Medicine, Bristol meeting, 183.
Library, Bristol Medical—L. M. Griffiths, 335.
Library, Bristol Medical, 371, 384.
Library of the Bristol Medico-Chirurgical Society, 93, 188, 283, 376 ; Removed to new premises, 192.
Local medical notes, 100, 199, 288, 383.

Malignant growths, The present position of the surgery of—C. A. Morton, 289.
Mastoid process, Surgical anatomy of, 97.
Medical organisation and the growth of the medical sciences in the seventeenth century—Dr. G. Parker, 201.
Medicine, Reports on—Dr. J. R. Charles, 345 ; Dr. J. M. Fortescue-Brickdale, 165 ; Dr. R. S. Smith, 67.
Mole, H. F.—Report on surgery, 348.
Morton, C. A.—Four cases of cysts in the neck of similar character, 160 ; The present position of the surgery of malignant growths, 289.

Neck, Cysts in the—C. A. Morton, 160.
Neild, Dr. N.—Some observations on tea-poisoning, 312.
Neuritis, herpes and—G. M. Smith, 194, 332.
Nixon, Dr. J. A.—Household syphilis, with observations on acquired syphilis in infants, 301.

Obituary, 98, 195, 284, 382.
Œdema, A family type of general, 192.
Ophthalmology, Report on—C. H. Walker, 77.

Parker, Dr. G.—Medical organisation and the growth of the medical sciences in the seventeenth century, illustrated by the lives of local worthies, 201.
Pneumococcal arthritis, 73.
Poisoning, Tea—Dr. N. Neild, 312.
Portal pyæmia, 191.
Posterior spinal nerve roots, Resection of the—E. W. H. Groves, 105, 191.
Pulmonary tuberculosis, Tuberculin in, 67.
Pulmonary tuberculosis, Compulsory notification of, 368.

Radiographic camera, Design for an elementary—Dr. W. Cotton, 118.
Radium treatment of tumour of cheek, 380.
Rheumatic infection, Histology of, 191.
Ribs, Cervical, 348.
Ringworm, An anomalous form of, 97.
Rogers, Dr. B. M. H.—The medical aspect of Boswell's *Life of Johnson*, 125.

Salvarsan in ophthalmology, 79.
Sanatoria and tuberculin dispensaries, 88.
School hygiene, Some observations on—Dr. J. O. Symes, 109, 193.
Seventeenth century, Medical organisation and the growth of the medical sciences in—Dr. G. Parker, 201.
Short, Dr. A. R.—The end-results of operations on the stomach and duodenum, 194, 220.
Shoulder-joint, Treatment of habitual dislocation of, 193.
**Sick, Preparations for the—**
Adalin, 373.
Ammonium chloride inhaler, 91.
British Medical Association exhibition, 281.
Chapman's entire wheat food, 186.
„ malted food, 187.
Colour photography, Tabloid chemicals for, 90.
Diphtheria antitoxin, Concentrated, 186.
Epinine, 90, 281.
Ferro-sajodin, 374.
Formalets, 375.
Glycero-phosphates, Huxley's, 375.
Gynoval, 374.
Hemisine and cocaine, 92.
Iodex, 375.
Junora, 282.
Palol, 375.
Palatinoid—filmaron, 187.
malt diastase, 374.
Pulverettes—calcium iodide, 187.
calcium permanganate, 187.
iodsam, 187.
Sea-water plasma, 187.
Soloid nizin, 186.
Sophol, 92.
Standard wholemeal biscuits, 187.
Streptococcus vaccine, rheumatic fever, 90.
Tabloid xaxa and caffeine, 281, 376.
Turin international exhibition, 185.
Valerobromine, 375.
Voebt's legumine biscuits, 282.

Smallpox amongst the Basutos, 279.
Smith, G. M.—Herpes and brachial neuritis, 194, 332.
Smith, Dr. L. S.—Furunculosis, 157.
Smith, Dr. R. S.—Report on medicine, 67.
Societies—Bristol Medico-Chirurgical Society, 95, 191, 378; British Medical Association, 278; Laryngological section of the Royal Society of Medicine, 183.
Spinal nerve roots, Resection of the posterior—E. W. H. Groves, 105, 191.
Stomach and duodenum, The end-results of operations on the—Dr. A. R. Short, 194, 220.
Stone in the bladder, Treatment of, 169.
Surgery of malignant growths, The present position of the—C. A. Morton, 289.
Surgery, Reports on—H. G. Kyle, 169; R. G. P. Lansdown, 73; H. F. Mole, 348.
Symes, Dr. J. O.—Some observations on school hygiene, 109, 193.
Syphilis, Cerebro-spinal—Dr. J. M. Clarke, 1.
Syphilis, Household—Dr. J. A. Nixon, 301.

Tea poisoning—Dr. N. Neild, 312.
Thompson, J. T., Obituary notice of, 195.
Thoracic duct, Wounds of the, 351.
Trachoma, 80.
Trypanosomiasis in man and animals, 95.
Tuberculin, Administration of, 345.
Tuberculin causing multiple arthritis, 348.
Tuberculin dispensaries, 88.
Tuberculin in pulmonary tuberculosis, 67.
Tuberculosis, Royal commission on, 280.
Tumour of a dog's tail, 97.
Tumour of cheek treated with radium, 380.

University of Bristol, 100, 101, 199, 288, 383.

Visual fields, Defects in the—F. R. Cross, 33.

Walker, C. H.—Report on ophthalmology, 77.
Williams, Dr. E. C.—A case of ataxia in a child, 163.
Wills, H. O., Obituary notice of, 287.
Word blindness, case of, 195.

### Bristol Medico-Chirurgical Society.

### PAST PRESIDENTS.

1874—75 FREDERICK BRITTAN, M.D., B.A. Dub.
1875—76. ROBERT WILLIAM COE.
1876—77. SAMUEL MARTYN, M.D. Ed.
1877—78. AUGUSTIN PRICHARD, M.D. Berlin.
1878—79. HENRY EDWARD FRIPP, M.D. Würzburg.
1879—80. WILLIAM MICHELL CLARKE.
1880—81. JOSEPH GRIFFITHS SWAYNE, M.D. Lond.
1881—82. EDWARD LONG FOX, M.D. Oxon.
1882—83. JAMES GEORGE DAVEY, M.D. St. And.
1883—84. WILLIAM JOHNSTONE FYFFE, M.D., B.A. Dub.
1884—85. GEORGE FORSTER BURDER, M.D. Aberd.
1885—86. WILLIAM HENRY SPENCER, M.D., M.A. Cantab.
1886—87. FRANCIS POOLE LANSDOWN.
1887—88. LEMUEL MATTHEWS GRIFFITHS.
1888—89. ROBERT SHINGLETON SMITH, M.D., B.Sc. Lond.
1889—90. NELSON CONGREVE DOBSON.
1890—91. SAMUEL HENRY SWAYNE.
1891—92. FRANCIS RICHARDSON CROSS, M.B. Lond.
1892—93. EDWARD MARKHAM SKERRITT, M.D., B.S., B.A. Lond.
1893—94. JAMES GREIG SMITH, M.B., C.M., M.A. Aberd., F.R.S.E.
1894—95. ALFRED JAMES HARRISON, M.B. Lond.
1895—96. ARTHUR WILLIAM PRICHARD.
1896—97. ALFRED EDWARD AUST LAWRENCE, M.D., C.M. Aberd.
1897—98. JOHN EDWARD SHAW, M.B., C.M. Ed.
1898—99. ROBERT ROXBURGH, M.B. Ed.
1899—00. WILLIAM HENRY HARSANT.
1900—01. DAVID SAMUEL DAVIES, M.D. Lond.
1901—02. BARCLAY JOSIAH BARON, M.B., C.M. Ed.
1902—03. GEORGE MUNRO SMITH.
1903—04. JAMES PAUL BUSH, C.M.G.
1904—05. REGINALD EAGER, M.D. Lond.
1905—06. JOHN DACRE.
1906—07. JAMES TAYLOR.
1907—08. HENRY WALDO, M.D., C.M. Aberd
1908—09. JOHN MICHELL CLARKE, M.D. Cantab.
1909—10. JAMES SWAIN, M.D., M.S. Lond.

1911.

### LIST OF SUBSCRIBERS

TO THE

### Bristol Medico-Chirurgical Journal.

*Those marked * are Members of the Society.*

| | |
|---|---|
| Ackland, J. McKno | 1 Barnfield Crescent, Exeter. |
| *Ackland, W. R. | 5 Rodney Place, Clifton. |
| Adye, W. J. A. | Church House, Bradford, Wilts. |
| Aldous, George F. | Charlton House, Mannamead, Plymouth |
| *Alexander, D.A., M.B., Ch.B. Vict. | 30 Berkeley Square, Clifton. |
| Ambrose, J., M.B. C.M. Aberd. | 138 Fishponds Road, Eastville, Bristol. |
| *Aubrey, T., M.B. | Bitton, near Bristol. |
| Aveline, H. T. S. | Somerset Asylum, Taunton. |

| | |
|---|---|
| *Ballance, H. Stanley, M.D. Lond. | 4 Royal Terrace, Weston-super-Mare |
| Barber, M. C. | Staple Hill, Bristol. |
| Baretti, T. G. L'Enardi | 49 Royal York Crescent, Clifton. |
| *Barker, G. H., M.B., B.Sc. Lond. | 124 Redland Road, Bristol |
| *Baron, Barclay J., M.B., C.M. Ed. | 16 Whiteladies Road, Clifton. |
| Basset, Walter | 24 Stow Hill, Newport, Mon. |
| Batten, W. Smith | West Hill, Calne. |
| Bayer Co., The | 19 St. Dunstan's Hill, London, E.C. |
| *Beddoe, John, M.D. Ed., B.A. Lond., F.R.S. | The Chantry, Bradford-on-Avon. |
| *Benson, J. | 11 The Circus, Bath. |
| *Bergin, F. G. | 12 West Park, Clifton. |
| Bernard, C. | 2 Spencer Terrace Fishponds. |

### LIST OF SUBSCRIBERS.

| | |
|---|---|
| *Berry, F. C., M.D., B.Ch., B.A. Dub. ... ... | Burnham, Somerset. |
| Berry, James, M.B., B.S. Lond. ... ... ... | 21 Wimpole Street, London, W. |
| Biamonti, Sebastiano ... ... ... ... ... ... ... | 27 Via Balbi, Genoa, Italy. |
| *Blacker, A. E.... ... ... ... ... ... ... ... ... | 20 Victoria Square, Clifton. |
| Blackett, J. F., M.B., B.S. Lond. ... ... ... | Audley, Newcastle, Staffs. |
| *Blacklfy, F. J., M.D. Dub. ... ... ... ... | 2 Knowle Road, Bristol. |
| *Blagg, Arthur F., M.D. Durh.... ... ... ... | 28 Caledonia Place, Clifton. |
| Bodman, Hervey, M.D. Lond. ... ... ... ... | 3 Whiteladies Road, Clifton. |
| *Boucher, F. T. ... ... ... ... ... ... ... ... | 31 Downleaze, Stoke Bishop. |
| *Bowker, G. E., M.D. Edin.... ... ... ... ... | 11 North Parade, Bath. |
| Boyce, James G. ... ... ... ... ... ... ... ... | The Chipping, Wotton-under-Edge. |
| Boyd, Geo. S. J. ... ... ... ... ... ... ... ... | 19 St. George's Square, Stamford, Lincs. |
| *Brasher, Charles W. J., M.B., Ch.B. Bristol | 128 Cotham Brow, Bristol. |
| *Brew, R. H. ... ... ... ... ... ... ... ... ... | Chew Magna. |
| *Bristowe, H. C., M.D. Lond. ... ... ... ... | Wrington, Somerset. |
| Brown, G. Arthur ... ... ... ... ... ... ... | Tredegar. |
| *Brown, William, M.D., C.M. Glasg. ... ... | Park View, Fishponds, Gloucestershire. |
| *Bullen, F. St. John ... ... ... ... ... ... ... | 3 Richmond Park Road, Clifton. |
| Burroughs, E. F. H. ... ... ... ... ... ... ... | 27 Burlington Road, Redland, Bristol. |
| *Bush, J. Paul, C.M.G. ... ... ... ... ... ... | Vyvyan House, Clifton Park, Clifton. |

| | |
|---|---|
| *Cadman, E. S. R., M.D., C.M. Edin. ... ... | 22 Trelawney Road, Cotham. |
| *Carling, A., M.A., M.B., B.C. Cantab. ... ... | 12 Great George Street, Bristol. |
| *Carter, T. M., M.D. Durh. ... ... ... ... | Burnley, Westbury-on-Trym. |
| *Carwardine, Thomas, M.B., M.S. Lond. ... | 16 Victoria Square, Clifton. |
| *Cave, Edward J., M.D. Lond. ... ... ... ... | 20 Circus, Bath. |
| *Charles, J. R., M.A., M.D., B.C. Cantab. ... | 12 Victoria Square, Clifton. |
| *Chitty, H., M.S. Lond. ... ... ... ... ... | Royal Infirmary, Bristol. |
| Chute, Henry M. ... ... ... ... ... ... ... ... | King William's Town, S. Africa. |
| *Clarke, C., M.B., B.S. Lond. ... ... ... ... | 17 Elmdale Road, Clifton. |
| *Clarke, John Michell, M.D., M.A. Cantab. | 28 Pembroke Road, Clifton. |
| Cobbledick, A. S., M.D., B.S. Lond. ... ... | 402 Brixton Road, London, S.W. |
| Coleridge, Alfred, M.B., B.S. Lond. ... ... | Cross Tree House, Moretonhampstead, Devon. |
| Connellan, Lieut. P. S., I.M.S. ... ... ... | |
| *Cook, Henri, M.D., C.M. Aberd. ... ... ... | 1 Dean Lane, Southville, Bristol. |
| *Coombs, Carey, M.D. Lond. ... ... ... ... | 74 St. Paul's Road, Clifton. |
| *Corfield, C. .. ... ... ... ... ... ... ... | 188 Wells Road, Knowle. |
| Cossham, W. R., M.D., C.M. Aberd.... ... ... | Cirencester. |
| Cotton, William, M.B., C.M., M.A. Ed. ... | 231 Gloucester Road, Bristol. |
| Coulter, R. J., M.B. Dub. ... ... ... ... ... | 11 Clytha Park Road, Newport, Mon. |
| Cridland, A. B. ... ... ... ... ... ... ... | 52 Waterloo Road, Wolverhampton. |
| *Cross, F. Richardson, M.B. Lond.... ... ... | Worcester House, Clifton. |
| *Crossman, F. W. ... ... ... ... ... ... ... | Hambrook, Gloucestershire. |
| *Crouch, C. Percival, M.B. Lond. ... ... ... | 1 Royal Terrace, Weston-super-Mare. |

| | |
|---|---|
| *Dacre, John ... ... ... ... ... ... ... ... ... | 14 Eaton Crescent, Clifton. |
| *Davies, D. S., M.D. Lond., D P.H. Cantab. ... | 60 Oakfield Road, Clifton. |
| *Davies, G. A. ... ... ... ... ... ... ... ... | 11 Alexandra Road, Clifton. |

### LIST OF SUBSCRIBERS.

| | |
|---|---|
| Dening, Edwin ... ... ... ... ... ... ... | Manor House, Stow-on-the-Wold. |
| Devine, H., M.D., Lond. ... ... ... ... ... | West Riding Asylum, Wakefield. |
| Dickie, James Steel, M.D., C.M. Aberd. ... | Boscombe Park, Bournemouth. |
| *Dickinson, W. G., M.D., Durh. ... ... ... | Wood Hill, Adelaide Terr., Portishead. |
| *Dixon, T. B. ... ... ... ... ... ... ... ... | Bristol Dispensary. |
| *Dobson, Nelson C. ... ... ... ... ... ... | 16 College Road, Clifton. |
| *Dowling, E. A. G. ... ... ... ... ... ... | 5 Rodney Place, Clifton. |
| Dunbar, Eliza L. Walker, M.D. Zürich ... | 9 Oakfield Road, Clifton. |
| Dymoke, F. ... ... ... ... ... ... ... ... | 21 Cotham Road, Bristol. |

| | |
|---|---|
| Eadon, W. F. Bailey ... ... ... ... ... ... | Hambrook, Gloucestershire. |
| *Eager, Reginald, M.D. Lond. ... ... ... ... | Northwoods, Winterbourne. |
| *Edgeworth, F. H., M.B., B.C., B.A. Cantab., D.Sc. Lond. ... ... ... ... ... ... ... ... | 4 Richmond Park Road, Clifton. |
| Edwards, E. S. ... ... ... ... ... ... ... | Empingham, Stamford. |
| Elder, William ... ... ... ... ... ... ... | 4 Trelawney Road, Bristol. |
| Elliot Bros. Ltd. ... ... ... ... ... ... | *Australian Pharmaceutical Notes & News* O'Connell Street, Sydney, N.S.W. |
| *Elliott, Christopher, M.D., B.A. Dub. ... | 3 Beaufort Road, Clifton. |
| Elliott, F. Percy, M.B., C.M. Aberd. ... ... | 113 Grove Road, Walthamstow, London N.E. |
| *Elliott, W. H. A., M.B., B.S. Lond. ... ... | Rockfort, Arley Hill, Bristol. |
| *Every-Clayton, L. E. V., M.D. Lond. ... ... | Rosslyn, Clevedon, Somerset. |

| | |
|---|---|
| *Fells, A., M.B., C.M. Edin. ... ... ... ... | 107 Ashley Road, Bristol. |
| *Ferguson, R. S., B.A. Durh., M.B. C.M. Edin. | Elm Grove, Calne, Wilts. |
| *Firth, J. L., M.D., M.S. Lond. ... ... ... ... | 8 Victoria Square, Clifton. |
| *Flemming, A. L., M.B., Ch.B. Bristol ... ... | 34 Alma Road, Clifton. |
| *Flemming, C. E. S. ... ... ... ... ... ... | Manvers House, Bradford-on-Avon. |
| *Fortescue-Brickdale, J. M., M.A., M.D., B.Ch. Oxon. ... ... ... ... ... ... ... ... | 52 Pembroke Road, Clifton. |
| Forty, D. H. ... ... ... ... ... ... ... ... | Wotton-under-Edge. |
| Foss, E. V. ... ... ... ... ... ... ... ... | Clouds Hill House, St. George, Bristol. |
| Fox, F. F. ... ... ... ... ... ... ... ... ... | Yate House, Yate. |
| *Fraser, Forbes ... ... ... ... ... ... ... | 2 The Circus, Bath. |
| Freeman, J., M.D. Brux. ... ... ... ... ... | Waterloo Villa, Queen's Road, Clifton. |

| | |
|---|---|
| Garland, E. B., M.B., C.M. Edin. ... ... ... | 114*a* Hampton Road, Redland. |
| Gee, C. A. H. ... ... ... ... ... ... ... ... | Evercreech, Somerset. |
| Genge, T. T. ... ... ... ... ... ... ... ... | 21 Whiteladies Road, Bristol. |
| *Gerrish, D. S. ... ... ... ... ... ... ... | 322 Church Road, St. George, Bristol. |
| *Gibbs, A. N. Godby ... ... ... ... ... ... | 52 Whiteladies Road, Clifton. |

| | |
|---|---|
| *Gratte, C. Brooke ... ... ... ... ... ... | 19 Gold Tops, Newport, Mon. |
| *Green, T. A., M.D., C.M. Edin. ... ... ... | 33*a* Whiteladies Road, Clifton. |
| Green-Armytage, Capt. V. B., I.M.S. ... ... | c/o King & Co., Bombay. |
| *Greer, W. J. ... ... ... ... ... ... ... ... ... | 19 Gold Tops, Newport, Mon. |
| *Grey, Harry, M.D., C.M.Edin. ... ... ... | 492 Fishponds Road, Bristol. |
| Griffiths, Cornelius A. ... ... ... ... ... ... | 6 Windsor Place, Cardiff. |
| *Griffiths, J. S. ... ... ... ... ... ... ... | 20 Redland Park, Bristol. |
| *Griffiths, L. M. ... ... ... ... ... ... ... ... | 11 Pembroke Road, Clifton. |
| *Groves, E. W. H., M.D., M.S. Lond. ... ... | 16 Richmond Hill, Clifton. |

| | |
|---|---|
| *Hailes, Clements D. G., M.D., C.M. Ed. ... | 27 Alma Road, Clifton. |
| *Hall, E. G., M.B. Lond. ... ... ... ... ... | 53 Hampton Road, Redland, Bristol. |
| *Hall, I. Walker, M.D., Ch.B.Vict. ... ... | 9 Royal Park, Clifton. |
| Handfield-Jones, Montagu, M.D. Lond. ... | 35 Cavendish Square, London, W. |
| *Harris, H. Elwin, B.A., M.B. Cantab. ... ... | 13 Lansdown Place, Clifton. |
| *Harsant, W. H. ... ... ... ... ... ... ... ... | Tower House, Clifton. |
| Hartnell, E. B. ... ... ... ... ... ... ... ... | 13 Church Street, Bridgwater. |
| *Hawes, I. H., M.B., B.S. Durh. ... ... ... | Wick, Glos. |
| Hayman, Alfred G. ... ... ... ... ... ... ... | Hapsford House, near Frome. |
| *Hayman, C. A., M.D. St. And. ... ... ... ... | Kingston Villa, Richmond Hill, Clifton |
| *Hele, T. S., M.A., M.B. ... ... ... ... ... ... | Royal Infirmary, Bristol. |
| *Herapath, C. E. K., M.D. Lond. ... ... ... | 12 Brunswick Square, Bristol. |
| *Herapath, C. K. C. ... ... ... ... ... ... ... | Cotham Park, Bristol. |
| Hill, Hedley ... ... ... ... ... ... ... ... ... | 1 Whiteladies Road, Clifton. |
| *Hill, Walter J. ... ... ... ... ... ... ... ... | The Brow, Clevedon. |
| Hospital, Bristol General (*Sec.*, W. Thwaites). | |
| Hospital, Taunton and Somerset (*House-Surgeon*, W. Drake). | |
| Huggard, W. R., M.D., M.Ch., M.A. Roy. Univ. ... ... ... ... ... ... ... ... ... | Davos-Platz, Switzerland. |

| | |
|---|---|
| *Iles, A. E., M.B., B.S. Lond. ... ... ... ... | Cotham Lawn, Cotham Park, Bristol. |
| *Imlay, G. A., M.B., C.M. Aberd. ... ... ... | 1 Cotham Park, Bristol. |
| Infirmary, Bristol Royal (*Secretary*, W. E. Budgett). | |
| Ingle, C. Durban ... ... ... ... ... ... ... ... | Somerton, Somerset. |
| *Irwin, S., M.B., B.Ch., B.A.O., R.U.I. ... ... | Olveston, Glos. |

| | |
|---|---|
| *James, C. W. W. ... ... ... ... ... ... ... ... | 246 Wells Road, Knowle. |
| Jamison, A., M.B., B.Ch., M.A. R.U.I. ... ... | Ardenvohr, Woodstock Road, Belfast. |
| *Jones, E. J. Trevor, M.D. Brux. ... ... ... | Aberdare, Glamorganshire. |
| *Jones, J. Ellington ... ... ... ... .. ... | 16 Richmond Park Road, Clifton. |

| | |
|---|---|
| JONES, H. MACNAUGHTON, M.D., M.Ch.R.U.I. | 131 Harley Street, London, W. |
| JOSEPH, A. HILL, M.D. Lond. ... ... ... ... | Bexhill-on-Sea. |

| | |
|---|---|
| *KEIR, I. C., M.D. Ed., D.P.H. ... ... ... ... | The Limes, Melksham. |
| *KEMM, F. ST. J.... ... ... ... ... ... ... ... | Worle. |
| *KYLE, H. G., M.B., B.Ch. Oxon. ... ... ... | 31 Westbury Road, Westbury-on-Trym. |

| | |
|---|---|
| *LACE, F. ... ... ... ... ... ... ... ... ... | 5 Gay Street, Bath. |
| *LANSDOWN, R. G. P., M.B., B.S. Dur. ... ... | 39 Oakfield Road, Clifton. |
| *LAVINGTON, C. C., M.B., B.S. Dur. ... ... | 1 Burlington Road, Redland. |
| LENDON, A. A., M.D. Lond. ... ... ... ... | North Terrace, Adelaide, S. Australia. |
| LE SOUDIER, H. ... ... ... ... ... ... ... | 174 et 176 Boulevard St. Germain, Paris. |
| *LEES, E. LEONARD, M.D., C.M. Ed. ... ... ... | 1 Chesterfield Place, Clifton. |
| LEIGH, W. W. ... ... ... ... ... ... ... ... | Treharris, Glamorganshire. |
| *LEONARD, R. C., M.D. Ed. ... ... ... ... ... | 134 Coronation Road, Bristol. |
| *LINDSAY, J., M.D. Ed. ... ... ... ... ... ... | 24 Bennett Street, Bath. |
| *LUCAS, J. J. S., M.D. Lond. ... ... ... ... ... | 186 Wells Road, Totterdown. |
| LUCY, REGINALD H. ... ... ... ... ... ... | 9 The Crescent, Plymouth. |

| | |
|---|---|
| *McCREA, P. W., M.D., B.Ch., B.A.O., B.A. Dub. ... ... ... ... ... ... ... ... ... ... | 19 Portland Square, Bristol. |
| MAC DONALD, P. W., M.D., C.M. Aberd.... ... | Herrison, Dorchester. |
| *MACKIE. Capt. F. P., I.M.S. ... ... ... ... | c/o Grindlay, Groom & Co., London. |
| MACKINNON, CHARLES, M.B., C.M., M.A. Glasg. | Davaar, Cirencester. |
| MACLEAN, EWEN J., M.D., C.M. Ed.... ... ... | 12 Park Place, Cardiff. |
| MACLEAN, J. CAMPBELL, M.B., C.M. Ed.... ... | Swindon House, Swindon, Wiltshire. |
| *MACLEOD, D., M.B., B.C. Ed. ... ... ... ... | 84 Redland Road, Bristol. |
| *MACWATTERS, J. C. .. ... ... ... ... ... ... | Almondsbury. |
| MARSH, O. E. BULWER ... ... ... ... ... ... | Parkdale, Newport, Mon. |
| MARSHALL, ARTHUR L., M.B., B.A. Cantab ... | 206 Upper Clapton Road, London, N.E |
| *MARTIN, E. F., M.B. Ed. ... ... ... ... ... | 7 Royal Terrace, Weston-super-Mare. |
| *MARTIN, R. C. ... ... ... ... ... ... ... ... ... | 3 Clifton Vale, Clifton. |
| *MARTYN, G. J. KING, M.D., B.C., B.A. Cantab. ... ... ... ... ... ... ... ... ... | 12 Gay Street, Bath. |
| MASON, S. B. ... ... ... ... ... ... ... ... | 12 Park Terrace, Pontypool. |
| MASON, WILLIAM ... ... ... ... ... ... ... | Sedgemoor Cottage, St. Austell, Cornwall. |
| MIALL, C. L. ... ... ... ... ... ... ... ... | Elm Hayes, Paulton, near Bristol. |
| *MOLE, HAROLD F. ... ... ... ... ... ... ... | 19 Mortimer Road, Clifton, Bristol. |

### LIST OF SUBSCRIBERS.

| | |
|---|---|
| *MOORE, C. A., M.B., M.S. Lond. ... ... ... ... | General Hospital, Bristol. |
| *MOORE, L. A., M.B., Ch.B., Bristol ... ... ... | 45 Ashley Road, Bristol. |
| MOORES, DE LA HEY ... ... ... ... .. ... | 6 West Mall, Clifton. |
| *MORGAN, T. WHITWORTH S. ... ... ... ... | The Laurels, Timsbury, Bath. |
| *MORTON, C. A. ... ... ... ... ... ... ... ... ... | 14 Vyvyan Terrace, Clifton. |
| *MUMFORD, W. G., M.B. Lond. ... ... ... ... | 18 The Circus, Bath. |
| MUNDEN, CHARLES ... ... ... ... ... ... ... | Ilminster. |
| *MUNRO, J. M. H., D.Sc. Lond. ... ... ... ... | 12 Grosvenor Place, Bath. |
| *MYLES, G T. ... ... ... ... ... ... ... ... | 7 Apsley Road, Clifton. |

| | |
|---|---|
| *NEILD, NEWMAN, M.B., B.Ch. Vict. ... ... ... | 9 Richmond Hill, Clifton. |
| *NEWMAN, HERBERT, M.B., B.S.Dur. ... ... | 150 Wells Road, Bristol. |
| *NEWNHAM, W. H. C., M.B., M.A. Cantab. ... | 3 Lansdown Place, Victoria Square, Clifton. |
| *NICHOLS, F. C., M.B., Ch.B. Bristol ... .. ... | 38 Whiteladies Road, Clifton. |
| NICHOLSON, T. D., M.D. Ed. ... ... ... ... | 2 Whiteladies Road, Clifton. |
| *NIXON, J. A., B.A., M.B., B.C. Cantab. ... ... | 19 Mortimer Road, Clifton. |
| NORMAN, J. H. ... ... ... ... ... ... ... ... | Goodleigh, Winkleigh, Devonshire. |

| | |
|---|---|
| ODELL, WILLIAM, M.D. Durh. ... ... ... ... | Ferndale, Torquay. |
| *OGILVY, ALEXANDER, M.D., B.Ch., B.A. Dub. | Leny, Clifton Park, Clifton. |
| OPENSHAW, E. H. ... ... ... ... ... ... ... ... | Cheddar. |
| OPPENHEIMER, SON & CO. LTD. ... ... .. | 179 Queen Victoria Street, E.C. |
| *ORMEROD, H. LAWRENCE, M.B., B.Ch. R.U.I. | 2 Henleaze Road, Westbury Park |
| *OWEN, SIR ISAMBARD, D.C.L., M.D. ... ... | Hereford House, Clifton. |

| | |
|---|---|
| PAGE, G. SHEPLEY ... ... ... ... ... ... ... | 78 Old Market Street, Bristol. |
| PAPILLON, J. W. ... ... ... ... ... ... ... ... | Brent Knoll, Somerset. |
| *PARKER, GEORGE, M.D., M.A. Cantab. ... ... | 14 Pembroke Road, Clifton. |
| PARKINSON, Lt. G. S., R.A.M.C. ... ... ... | Military Hospital, Belfast. |
| *PARSONS, H. F.... ... ... ... ... ... ... ... | The Heath, Stoke Bishop. |
| PARSONS, J. H., M.B., B.S., D.Sc. Lond.... ... | 27 Wimpole Street, London, W. |
| PATERSON, DONALD ROSE, M.D., C.M. Ed. ... | 18 Windsor Place, Cardiff. |
| PEAKE, ARTHUR W.... ... ... ... ... ... ... | 34 Gloucester Road, Bishopston, Bristol. |
| PEAKE, G. ARTHUR ... ... ... ... ... ... ... | Rodney Place, Cheltenham. |
| *PEARSE, J., M.D., C.M. Ed.... ... ... ... ... | 28 St. George's Terrace, Trowbridge. |
| PERDRAU, J. A., M.B. Lond. ... ... ... ... | 99 Gower Street, London, W.C. |
| *PETERS, B. A. I., M.B. Cantab. ... ... ... ... | Ham Green, Somerset. |
| PHILLIPS, C. M., M.D. Brux. ... ... ... ... | Ravenslea, Kensington Hill, Brislington. |
| *PICKERING, C. F. ... ... ... ... ... ... ... | 13 Berkeley Square, Bristol. |
| *PINNIGER, W. J. H., M.D., B.S. Lond. ... ... | 75 Effingham Road, Bristol. |
| *PORTEOUS, H. B., M.B., Ch.B. Ed. ... ... ... | Newbury House, Weston-super-Mare. |
| POWELL, J. J., M.D. Lond. ... ... ... ... ... | 2 Gloucester Road, Bishopston, Bristol. |
| POWELL, WILLIAM, M.B. Lond. ... ... ... ... | Hill Garden, Torquay. |
| PRICE, D. T. M.B. Lond. ... ... ... ... ... | Castle Cary, Somerset. |

### LIST OF SUBSCRIBERS.

| | |
|---|---|
| *PRICHARD, ARTHUR W. ... ... ... ... ... ... | 6 Rodney Place, Clifton. |
| *PROWSE, ARTHUR B., M.D. Lond. ... ... ... | 5 Lansdown Place, Clifton. |
| PROWSE, W. B.... ... ... ... ... ... ... ... | 31 Vernon Terrace, Brighton. |

| | |
|---|---|
| RANDOLPH, CHARLES ... ... ... ... ... ... | St. Michael's, Milverton, Somerset. |
| *RATTRAY, J. M., M.D., C.M., M.A. Aberd. ... | Frome. |
| *RAYNER, D. C. ... ... ... ... ... ... ... ... | 9 Lansdown Place, Clifton, Bristol. |
| RENDALL, JOHN ... ... ... ... ... ... ... | Forestside, Lymington. |
| RITCHIE. THOMAS P. ... ... ... ... ... ... | 27 Oakfield Road, Clifton. |
| *ROBERTSON, D., M.B., Ch.B. Edin. ... ... ... | Bristol Dispensary. |
| *ROGERS, B. M. H., M.D., B.Ch., B.A. Oxon. ... | 1 Victoria Square, Clifton. |
| *ROLFE, R., B.A., M.B., B.C. Cantab. ... ... | Park House, Avonmouth. |
| *ROSE, F. H. ... ... ... ... ... ... ... ... | 13 Westfield Park, Clifton. |
| *ROSSITER, GEORGE F., M.B. Lond. ... ... | Cairo Lodge, Weston-super-Mare. |
| *ROXBURGH, ROBERT, M.B. Ed. ... ... ... | 1 Victoria Buildings, Weston-super-Mare |
| *RUDGE, C. KING ... ... ... ... ... ... ... | 145 Whiteladies Road, Bristol. |
| *RUTHERFORD, J. M., M.B., C.M. Edin. ... ... | Brislington House, Bristol. |

| | |
|---|---|
| *SAVAGE, W. G., M.D. Lond., D.P.H. ... ... | Hafton, Uphill Road, Weston-s.-Mare. |
| *SCHÖLBERG, H. A., M.B., B.S. Lond. ... ... | University College, Cardiff. |
| SCOTT, BATY- ... ... ... ... ... ... ... | 28 Belluton Road, Knowle, Bristol. |
| SEWARD, W. J., M.B. Lond. ... ... ... ... | Colney Hatch Asylum, New Southgate, London, N. |
| *SHAW, J. E., M.B., C.M. Ed. ... ... ... ... | 23 Caledonia Place, Clifton. |
| SHEPPARD, Lieut. A. L., I.M.S., M.B., B.S. Dur. | c/o Grindlay, Groom & Co., Bombay. |
| *SHEPPARD, E. J. .. ... ... ... .. ... ... | 46 St. Paul's Road, Clifton. |
| *SHORT, A. R., M.D., B.S., B.Sc. Lond. ... ... | 2 Westbourne Place, Queen's Road, Clifton. |
| *SHORT, L. J., M.D., B.Sc. Lond. ... ... ... | 93 Stamford Hill, London. |
| SKELTON, HENRY, M.D. St. And. ... ... ... | Downend, Gloucestershire. |
| SMITH, Capt. F. SHINGLETON, I.M.S. ... ... | c/o Grindlay & Co., Bombay. |
| *SMITH, G. COCKBURN, M.D. Brux. ... ... ... | 14 South Road, Newton Abbot. |
| *SMITH, G. MUNRO ... ... ... ... ... ... ... | 18 Apsley Road, Clifton. |
| SMITH, L. SHINGLETON, B.A., M.B. Cantab.... | Honddu House, Brecon. |
| SMITH, REV. REGINALD, M.A. Cantab. ... ... | The Vicarage, Walpole St. Andrew, Norfolk. |
| *SMITH, R. SHINGLETON, M.D., B.Sc. Lond. ... | Clifton Park, Clifton. |
| SMITH, W. Steele, M.B. Lond. ... ... ... ... | 1 Wilbraham Rd., Chorlton-cum-Hardy, Manchester. |
| *SMITH, W. ALEXANDER, M.B., M.A. Oxon. ... | 70 Pembroke Road, Clifton. |

SOCIETY, CARDIFF MEDICAL (*Hon. Lib.*, Queen Street, Cardiff).

| | |
|---|---|
| SOCIETY, MANCHESTER MEDICAL ... ... ... | Medical Society's Library, The Owens College, Manchester. |
| SOCIETY, PLYMOUTH MEDICAL (*Hon. Lib.*, J. ELLIOTT SQUARE). | |
| *STACK, E. H. E., B.A., M.B., B.C. Cantab. ... | Arvalee, Clifton Down Road, Clifton. |
| STAPLE, J. D. ... ... ... ... ... ... ... ... | Clevedon Villa, Cheltenham Road, Bristol. |
| STATHAM, R. WHITESIDE ... ... ... ... ... | Cheddar, Somersetshire. |
| *STEELE, CHARLES, M.D. Durh. ... ... ... ... | 17 Richmond Hill, Clifton. |
| STEVENS, W. H. ... ... ... ... ... ... ... ... | 26 Zetland Road, Bristol. |
| STOCK, P. G., M.B., Ch.B. Bristol ... ... ... | Box 1049, Johannesburg, South Africa. |
| *STOCK, W. S. V., M.B., B.S. Lond. ... ... ... | Montrose House, Queen's Road, Clifton |
| STODDART, F. WALLIS ... ... ... ... ... ... ... | Southey House, College Green, Bristol. |
| *SWAIN, JAMES, M.D., M.S. Lond. ... ... ... | 4 Victoria Square, Clifton. |
| *SWAYNE, W. CARLESS, M.D. Lond. ... ... ... | St. Paul's Road, Clifton. |
| *SYMES, J. O., M.D. Lond. ... ... ... ... ... | 71 Pembroke Road, Clifton. |
| SYMONDS, H. P. ... ... ... ... ... ... ... | 35 Beaumont Street, Oxford. |

| | |
|---|---|
| TAYLER, H. P., M.B., B.C. Cantab. ... ... ... | The Abbey House, Bradford-on-Avon. |
| TAYLOR, A. L. ... ... ... .. ... ... ... ... | 22 Downfield Road, Clifton. |
| TAYLOR, C. J. ... ... ... ... ... ... ... ... | 43 Duke Street, Chelmsford. |
| *TAYLOR, JAMES ... ... ... ... ... ... ... ... | 36 Alma Road, Clifton. |
| *TAYLOR, J. W., M.B., Ch.B. Bristol ... ... ... | 8 West Redcliffe Parade, Bristol. |
| *TEMPLE, G. H., M.B., C.M. Ed. ... ... ... | Ailanthus, Weston-super-Mare. |
| THIN, JAMES ... ... ... ... ... ... ... ... | 54 & 55 South Bridge, Edinburgh. |
| THOMAS, A. GARROD, M.D., C.M. Ed. ... ... | Clytha Park, Newport, Mon. |
| *THOMAS, J. D., M.B. Cantab. ... ... ... ... | Northwoods, Winterborne, |
| THOMAS, J. LYNN, C.B. ... ... ... ... ... ... | 21 Windsor Place, Cardiff. |
| *THOMAS, J. M. M. ... ... ... ... ... ... ... | 5 Colston Parade, Redcliffe, Bristol. |
| *THOMAS, J. TUBB, D.P.H. .. ... ... ... ... | The Halve House, Trowbridge. |
| *THOMAS, R. E. M.B., B.S. Lond. ... ... ... | Royal Infirmary, Bristol. |
| THOMSON, EUSTACE B., M.D., C.M. Glasg. ... | Albany Place, Plymouth. |
| *THOMSON, F. G., M.A., M D. Lond. ... ... ... | 20 Gay Street, Bath. |
| TOSSWILL, LEONARD R., D.P.H., Cantab. ... | Devon County Council Offices, 14 Bedford Circus, Exeter. |
| TYLEY, R. P., M.D. St. And.... ... ... ... ... | Wedmore. |

| | |
|---|---|
| VACHELL, HERBERT R., M.D., C.M. Aberd. ... | 18 Newport Road, Cardiff. |
| *VICKERY, W. H. ... ... ... ... ,,, ... ... | 1 Trewartha Park, Weston-super-Mare. |

| | |
|---|---|
| *WALDO, HENRY, M.D., C.M. Aberd. ... ... ... | 19 Pembroke Road, Clifton. |
| *WALKER, CYRIL H., M.B., B.A. Cantab. ... ... | 8 Oakfield Road, Clifton. |
| WALLACE, REV. CANON, M.A. Oxon. ... ... ... | 3 Gloucester Row, Clifton. |
| *WALLACE, JOHN ... ... ... ... ... ... ... | Lauriston, 15 Knightstone Road, Weston-super-Mare. |
| WALLACE, J. T., M.B., B.Ch., B.A. R.U.I. ... | 48 Coronation Road, Bedminster. |
| *WALLACE, JAMES W. ... ... ... ... ... ... | 1 Greville Road, Southville, Bristol. |
| *WALLIS, G. F. C., M.B., Ch.B. Ed. ... ... ... | 16 Swan Hill, Shrewsbury. |
| *WALTERS, C. F. ... ... ... ... ... ... ... ... | 19 Whatley Road, Clifton. |
| *WATERHOUSE, R., M.D. Lond. ... ... .. ... | 28 Gay Street, Bath. |
| WEBBER, WILLIAM W. ... ... ... ... ... ... | Crewkerne. |
| *WHICHER, A. H. ... ... ... ... ... ... ... | 115 Queen's Road, Clifton |

| | |
|---|---|
| WHITE, C. R. ... ... ... ... ... ... ... ... | Nixon Villa, Merthyr Vale, Glamorgan. |
| WHITE, J. W. ... ... ... ... ... ... ... ... ... | Orchard House, Nailsea. |
| *WHITE, PERCY W. ... ... ... ... ... ... ... | College Green, Bristol. |
| WIGMORE, J., M.D. R. U. I.... ... ... ... ... | 16 Queen Square, Bath. |
| WILCOX, HENRY, M.B. Aberd. ... ... ... ... | 10 Willington Road, Eastbourne. |
| WILLCOX, R. LEWIS ... ... ... ... ... ... ... | Warminster. |
| WILLETT, GEORGE G. D. ... ... ... ... ... ... | Keynsham, Somerset. |
| *WILLIAMS, E. C., M.B., B.C., B.A. Cantab. ... | 159 Whiteladies Road, Clifton. |
| WILLIAMS, H. EGERTON ... ... ... ... ... | The Mount, Newport, Mon. |
| *WILLIAMS, P. WATSON, M.D. Lond.... ... ... | 2 Rodney Place, Clifton. |
| WILLIAMS, S. R., M.B. Lond. ... ... ... ... | Buckfastleigh, Devon. |
| *WILLIAMS, W. ROGER ... ... ... ... ... ... | Morven, Walton-by-Clevedon. |
| *WILLIAMSON, G. SCOTT... ... ... ... ... ... | Bristol General Hospital. |
| WILLIS, W. M.... ... ... ... ... ... ... ... ... | 7 Regent Street, Nottingham. |
| *WILLS, W. K., M.B., B.C. Cantab. ... ... ... | 19 Whiteladies Road, Clifton. |
| *WINDSOR-AUBREY, H. W. ... ... ... ... ... | Coronation Road, Bristol. |
| *WOOD, W. V. ... ... ... ... ... ... ... ... ... | Glencairn, Wrington. |
| WOOLLCOMBE, WALTER L. ... ... ... ... ... | 16 The Crescent, Plymouth. |
| *WRIGHT, A. J., M.D., B.S. Lond. ... ... ... | 60 St. Paul's Road, Clifton. |
| | |
| YOUNG, J., M.D. Glas. ... ... ... ... ... ... | 14 Chantry Road, Clifton. |

